# Effectiveness of a Simple Lymphoedema Treatment Regimen in Podoconiosis Management in Southern Ethiopia: One Year Follow-Up

**DOI:** 10.1371/journal.pntd.0000902

**Published:** 2010-11-30

**Authors:** Catherine Sikorski, Meskele Ashine, Zewdie Zeleke, Gail Davey

**Affiliations:** 1 University College London Medical School, London, United Kingdom; 2 Mossy Foot Treatment and Prevention Association, Wolaita Sodo, Ethiopia; 3 School of Public Health, Addis Ababa University, Addis Ababa, Ethiopia; Queensland Institute for Medical Research, Australia

## Abstract

**Background:**

Podoconiosis is a non-filarial elephantiasis caused by long-term barefoot exposure to volcanic soils in endemic areas. Irritant silicate particles penetrate the skin, causing a progressive, debilitating lymphoedema of the lower leg, often starting in the second decade of life. A simple patient-led treatment approach appropriate for resource poor settings has been developed, comprising (1) education on aetiology and prevention of podoconiosis, (2) foot hygiene (daily washing with soap, water and an antiseptic), (3) the regular use of emollient, (4) elevation of the limb at night, and (5) emphasis on the consistent use of shoes and socks.

**Methodology/Principal Findings:**

We did a 12-month, non-comparative, longitudinal evaluation of 33 patients newly presenting to one clinic site of a non-government organization (the Mossy Foot Treatment & Prevention Association, MFTPA) in southern Ethiopia. Outcome measures used for the monitoring of disease progress were (1) the clinical staging system for podoconiosis, and (2) the Amharic Dermatology Life Quality Index (DLQI), both of which have been recently validated for use in this setting. Digital photographs were also taken at each visit. Twenty-seven patients completed follow up. Characteristics of patients completing follow-up were not significantly different to those not. Mean clinical stage and lower leg circumference decreased significantly (mean difference -0.67 (95% CI −0.38 to −0.96) and −2.00 (95% CI −1.26 to −2.74), respectively, p<0.001 for both changes). Mean DLQI diminished from 21 (out of a maximum of 30) to 6 (p<0.001). There was a non-significant change in proportion of patients with mossy lesions (p = 0.375).

**Conclusions/Significance:**

This simple, resource-appropriate regimen has a considerable impact both on clinical progression and self-reported quality of life of affected individuals. The regimen appears ideal for scaling up to other endemic regions in Ethiopia and internationally. We recommend that further research in the area include analysis of cost-effectiveness of the regimen.

## Author Summary

Podoconiosis is a type of leg swelling that occurs in the tropics among subsistence farmers who spend long hours in contact with irritant soil derived from volcanic parent rock. People with this disease often live in very remote areas far from health facilities, and do not realize that the condition can be treated. A patient association in southern Ethiopia has pioneered low cost treatment. We followed 27 patients with podoconiosis through their first year of treatment. The regimen emphasizes understanding the disease process and self-treatment through foot hygiene, elevation of the limb at night, use of emollients, socks and shoes. We made clinical observations, measured quality of life and took digital photographs at the start and after 12 months of self-treatment. Leg circumference decreased by an average of 2 cm, the stage of disease decreased (indicating improvement) by almost one full stage. Quality of life improved dramatically, with patients reporting levels of life quality equivalent to non-patients. Even in a very resource-scarce setting, we have shown that patients benefit physically and psychologically from understanding the disease and being shown how to look after their feet.

## Introduction

Podoconiosis is a non-filarial lower-leg lymphoedema caused by long-term barefoot exposure to volcanic soils in endemic areas[1]. It has been described in equatorial countries across Africa, Central and South America and South Asia and was formerly noted in Europe before footwear became commonplace[2]. Irritant silicate particles in areas of high altitude (over 1000 m) and annual rainfall (over 1000 mm) penetrate the skin causing a progressive obliterative endolymphangitis[3]. The effect is a debilitating lymphoedema of the lower leg, with or without skin changes (hyperkeratosis and ‘mossy’ papillomata) and fibrotic nodule formation[4].

Unlike in lymphatic filariasis (LF)[5], podoconiosis lymphoedema appears to affect men and women equally[6]. A cross-sectional survey of an endemic region in Southern Ethiopia estimated a prevalence of 5.46% in this area, which, projected to the population now living on irritant soil in Ethiopia, suggests that one million Ethiopians may be affected[6]. The economic burden of the condition is considerable, with a loss of productivity equivalent to 45% of working days per patient annually[7]. The condition is poorly understood by the lay and medical communities alike, often being mistaken for infective filarial (Bancroftian) elephantiasis[8]. Affected individuals find themselves socially stigmatised, excluded from school, churches and mosques and unable to marry into an unaffected family[9].

A simple affordable treatment approach appropriate for resource poor settings has been developed, and shares many aspects of the management of LF lymphoedema[10], [11]. The regimen comprises (1) training in the aetiology of podoconiosis and the rationale behind secondary preventative measures, (2) foot hygiene, involving daily washing with soap, water and dilute bleach (acting as an antiseptic), (3) the regular use of emollient and/or antifungal (eg Whitfield's ointment), (4) elevation of the limb at night, and (5) emphasis on the importance of consistent use of shoes and socks[4]. In LF, foot hygiene is effective through diminishing bacterial and fungal infection of the swollen leg, through restoring skin integrity and by decreasing the frequency of acute adenolymphangitis attacks[10]. For podoconiosis patients, these mechanisms are also likely to be important, and in addition, washing and the use of shoes will lessen exposure to the environmental trigger.

Another important aspect of the treatment programme in southern Ethiopia is educational and social support, in the form of monthly meetings at which messages on prevention and treatment are given, and social and spiritual support are offered. Although evaluations of similar management regimens have been reported in relation to filarial lymphoedema[5], [12], no formal study has yet been conducted to gather evidence on effectiveness among podoconiosis patients. We aimed to test the effectiveness of a community-based, patient-led treatment regimen used in southern Ethiopia prior to scale-up of treatment efforts to other endemic regions in Ethiopia and internationally.

## Methods

### Ethics Statement

This study was conducted according to the principles expressed in the Declaration of Helsinki. The study was approved by the School of Public Health Research Ethics Committee, Addis Ababa University. All patients provided informed written or thumbprint consent for their data to be analysed. The Research Ethics Committee approved the use of thumbprint consent (countersigned by a witness) among illiterate patients, who constituted a small minority of participants.

We carried out a 12-month single-centre, non-comparative study to evaluate the treatment of newly diagnosed individuals enrolling onto a podoconiosis management regimen in Shanto Town, Wolaita Zone. This podoconiosis-endemic zone in southern Ethiopia[6] is a densely populated area, where most of the population earn a living from small-scale subsistence farming. The management regimen evaluated forms a key part of the work of the Mossy Foot Prevention and Treatment Association (MFPTA), a non-governmental organisation which has been active in the area for 10 years and currently works with over 30,000 patients[13].

New patients presenting to the clinic on a single day in June 2008 formed our cohort under study. Inclusion criteria were a clinical assessment of non-filarial elephantiasis (clinical assessment in this endemic area has high validity in exclusion of filarial disease[14]), and age above 18 years. Patients were excluded from the study if they were expecting to move out of the area during the study period (June 2008 to June 2009) or had previously been on a treatment programme for podoconiosis. Consent for participation in the study was sought from patients satisfying these criteria.

Baseline data were recorded at the initial clinic visit and treatment started immediately. Follow-up was then undertaken at 3-month intervals when the patients were established on the treatment programme, for a total period of one year (until June 2009). Outcome measures used for the monitoring of disease progress were (1) the clinical staging system for podoconiosis and (2) the Amharic Dermatology Life Quality Index (DLQI). Measures were recorded blind to previous measurements, by the same team of fieldworkers (supervised by CS, ZZ and MA). Compliance was evaluated using the proxy of attendance at interim clinic sessions (at which intervention items including soap, bleach and ointment, are distributed).

The clinical staging system for podoconiosis was modified from the Dreyer staging system for filarial lymphoedema[11] modified after it became clear that the disease progression of filarial and podoconiosis lymphoedema are quite different. This staging system has shown good content validity and good inter-observer agreement and repeatability in the clinical assessment of podoconiosis[15]. The Tekola clinical staging system for podoconiosis incorporates three clinical measures – numerical stage, presence (M+) or absence (M−) of mossy skin changes, and a measurement (in cm) of the widest below-knee circumference. These elements are then combined and recorded, for example “Stage 3, M+, 34” (see Supporting Text S1, Tekola Staging System, for more details). Limb measurements were made between 10am and 11am during clinic visits.

For the assessment of Quality of Life of affected individuals, the Cardiff Dermatology Life Quality Index (DLQI)[16] translated into Amharic, has recently been shown to have high internal consistency and concurrent validity among podoconiosis patients[17]. The ten questions which form the DLQI questionnaire explore diverse aspects of quality of life, including pain, embarrassment and interference with work and relationships. The final question raises the issue of problems caused by the treatment itself. A higher score indicates poorer quality of life. Digital photographs were also taken at each visit. Patients who did not attend follow-up clinics were visited and assessed at home by the same team of fieldworkers.

The data were analysed using the SPSS statistical package, version 11.0. For the analysis, M+/M− were given binary values; all other variables retained their original numerical value. Paired 12-month differences were calculated for the four variables (3 staging variables plus DLQI score) and checked for distribution and skewness. The significance of the mean difference in the numerical stage of disease, the presence or absence of mossy changes, the circumference measurement and the DLQI score were calculated using the sign test, McNemar's test, a paired t test and the sign test respectively. Ninety-five percent confidence intervals (95% CIs) were calculated for the mean difference in stage, leg circumference and DLQI score.

## Results

33 new patients presenting at Shanto Health Centre all satisfied the criteria for inclusion in the study and agreed to take part. Treatment started immediately and patients were followed up between June 2008 and June 2009. During this time 6 patients moved out of the area and were not included in our final analysis. Baseline characteristics of these patients were not significantly different to those who completed follow-up. Of the 27 patients followed up, 81.5% were female. While aware that ages given are often approximate, the age range of the sample was 18–60 years (mean 37.4 years, median 36 years). Thirty-seven percent had at least one affected family member. Compliance among the 27 followed up was good, only 3 patients missing one interim clinic visit, and no patient missing more than this.

### Clinical & DLQI Measurements at Baseline

Measurements taken at the first visit suggest a considerable burden of disease in this cohort (Table 1). Over 80% of patients presented with numerical disease stage of 2 or more, with mean stage for the cohort of 2.07. One-third displayed mossy changes, and leg circumference ranged from 20.0 to 35.5 cm (mean 26.22 cm, median 26.00 cm). Mean DLQI score was 21.11 (median 22.00, range 15–24) out of a possible total of 30.

**Table 1:** Changes in clinical variables and DLQI from baseline to 12 months.

	0 months	12 months	Mean difference (95% CI)	p-value
**Clinical Stage Mean**	2.07	1.41	−0.67	<0.001
**Clinical Stage Range**	1 to 3	1 to 3	−0.38 to −0.96	
**Circumference mean (cm)**	26.22	24.22	−2.00	<0.001
**Circumference range (cm)**	20 to 35	19 to 31	−1.26 to −2.74	
**DLQI Mean**	21.11	6.07	−15.04	<0.001
**DLQI Range**	15 to 24	0 to 13	−13.32 to −16.76	
			**Number (%) losing/gaining moss**	
**Moss present (%)**	9 (33.3)	6 (22.2)	4 (14.8)/1 (3.7)	0.375

DLQI  =  Dermatology Life Quality Index.

CI – Confidence Interval.

### Clinical & DLQI Measurements after 12 Months

After 12 months, 63.0% of patients were recorded as having stage 1 disease with only one patient remaining at stage 3, compared to seven at the beginning of the treatment regimen. Just over one fifth (22.2%) still had mossy changes, while leg circumference range had decreased to 19.0–31.0 cm (mean 24.22 cm, median 24.00 cm). The most marked change was in mean DLQI score, which had decreased to 6.07 (median 5.00, range 0–13) out of a possible maximum of 30. Three patients (11.1%) reported a DLQI score of 0, suggesting a quality of life unaffected by their condition.

### Changes in Measurements Between Baseline and 12 Months

**Figure 1:**
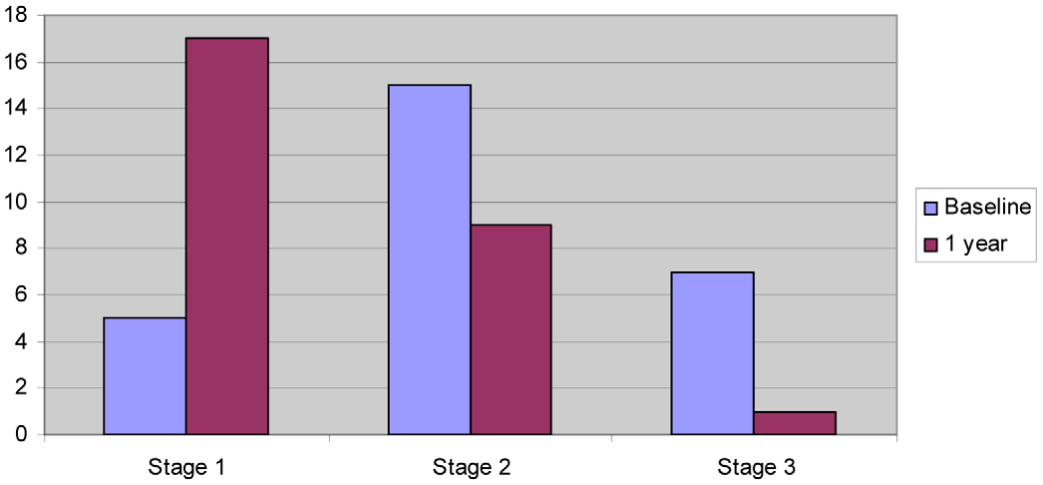
Numbers of patients by clinical stage at baseline and 12 months.

Analysis of the changes in variables from 0 to 12 months of treatment largely confirms the statistical significance of our findings. Clinical stage decreased in 51.9% of patients and increased in none (mean decrease 0.67, p<0.001, 95% CI 0.38 to 0.96). Changes in clinical stage from baseline to one year are shown graphically in Figure 1. Mean reduction in leg circumference was 2.00 cm (p<0.001, 95% CI 1.26 to 2.74), with 85.2% of patients showing a decrease of at least 1 cm. Every patient reported a reduced DLQI score and for 96.3% of patients this was a change of 10 points or more. Mean decrease in DLQI score was 15.04 (p<0.001, 95% CI −13.32 to −16.76). The only variable not showing a statistically significant treatment effect after 12 months was the presence of mossy changes. Four patients had lost and one patient gained mossy lesions, with a p-value for these changes of 0.375.

## Discussion

This simple, resource-appropriate, lymphoedema management regimen is effective in improving clinical outcomes and self-reported quality of life of people affected with podoconiosis.

Before discussing the improvements observed in comparison with other studies, the limitations of this follow-up study must be acknowledged. The most important of these is the lack of an untreated control group. In common with other groups faced with evaluating lymphoedema management regimens already adopted by communities (LeAnne Fox, personal communication), we found it ethically difficult to deny affected individuals access to the regimen because of strong observational evidence in favour of the treatment. Podoconiosis lymphoedema has never been reported as regressing of its own accord, making it likely that the improvements observed are attributable to the management regimen. This small, observational pilot study suggests the need to conduct a formal randomized controlled trial of lymphoedema management among podoconiosis patients. This might be achieved ethically by randomizing patients or communities to ‘immediate’ or ‘delayed’ treatment, with outcomes compared between groups after one year, prior to starting treatment in the ‘delayed’ group. Several organizations, government and non-government, are planning introduction of podoconiosis control measures in endemic areas of Ethiopia in which no treatment is currently available, and the initial stages might provide ideal settings for a trial of this type.

It is possible that the participants followed were to some extent self-selecting, and may therefore represent those with a higher level of commitment to treatment than the entire podoconiosis population from which they came. As this was a piece of operational research, however, it was important not to alter the process of selection already in place. It is difficult to know whether the gender imbalance of the sample affected our findings. Subgroup analysis by sex showed slightly greater changes in all variables for men than for women, but none of these comparisons was statistically significant.

Loss to follow-up was experienced in 6/33 (18%) of the cohort. Comparison of demographic and clinical characteristics between those completing follow up and those lost to follow up did not suggest significant differences in these factors among those lost to follow up, thus bias related to follow-up is unlikely to have been introduced.

In order to minimise observer bias, fieldworkers who recorded both clinical stage and DLQI were blind to the patients' previous scores. Level of compliance (regularity of emollient application, washing, elevation and shoe use) was not monitored, limiting the extent to which we can measure a ‘dose-effect’ of the treatment. In particular, availability of water to wash with was not ensured by the program, and during the final 4 months of self-treatment (towards the end of the dry season), some patients reported this to be a limiting factor. This evaluation is therefore highly pragmatic, representing podoconiosis treatment as implemented in the ‘real world’ in rural Ethiopia.

After twelve months of treatment, we observed small but significant changes in clinical outcomes. Clinical stage decreased in more than half of patients, with a mean decrease of 0.67 stage. The Tekola clinical staging system has been shown to have high inter-observer agreement and to be highly repeatable over 1 week[15], but it is possible that the fieldworkers were influenced by a form of ‘social desirability’ bias when presented with borderline cases, resulting in recording of lower disease stage at 12 months. Lower leg circumference is a less subjective measure, and decreased on average by 2 cm or 7.5% of the original. We did not measure leg volume, but extrapolation from a 2 cm decrease in circumference suggests a clinically as well as statistically significant change. We were unable to take biopsies in this very remote rural setting, and have no information on reversal of pathological changes associated with these clinical changes. Addiss's group in Haiti studied the effectiveness of 12 months lymphoedema management on histological markers of chronic inflammation in LF patients. They demonstrated distinct improvements (reduction in perivascular and periadnexal infiltrates, reduction in deep dermal fibrosis) in the 27 patients studied[5].

Initial average quality of life score, at 21 units, was higher than the average recorded (13 units) among previously untreated patients at another treatment site in 2007[17], or among filarial lymphoedema patients using the modified DLQI (8 units[18]). At 15 units the average decrease in DLQI over 12 months was larger than the difference between treated and new podoconiosis patients (10 units[17]). It is unclear to what extent this is due to tangible clinical change, rather than simply a consequence of the social and community aspects of the intervention. A similar study, reporting the effect of lymphoedema management on quality of life among 15 lymphatic filariasis patients in Guyana also showed significant decrease in DLQI. Mean DLQI fell from 10.9 to 4.1 (p<0.0001), and the author suggested several possible reasons for this improvement, including increased ability to work and perform daily functions, empowerment in relation to the condition and its management, and ability to network with other patients[12].

No previous follow up studies of lymphoedema management in podoconiosis have been reported. We recommend that further research in the area could include analysis of the cost-effectiveness of the programme, establishing a treatment cost per unit improvement in each variable. Also currently lacking is a qualitative exploration of patients' perceptions of the treatment and its key elements. As the programme is introduced to new areas it will also be important to assess its impact with ‘before and after’ trials. The implications of our findings have the potential to be far-reaching. This simple patient-led approach is feasible even in very remote rural areas, and appears ideal for scaling up to other podoconiosis-endemic regions in Ethiopia and internationally. The regimen has considerable overlap with that suggested for control of LF morbidity[10], so we recommend that expansion of podoconiosis control is integrated with that of LF control in countries where both forms of lymphoedema contribute significantly to morbidity.

## Supporting Information

Checklist S1 Strobe Checklist
(0.09 MB DOC)

Text S1 Tekola Staging System
(0.03 MB DOC)
