# Supplementary material for: Effectiveness of a Simple Lymphoedema Treatment Regimen in Podoconiosis Management in Southern Ethiopia: One Year Follow-Up
Source: PLoS Negl Trop Dis. 2010 Nov 30;4(11):e902. doi: 10.1371/journal.pntd.0000902 (PMC2994920; doi:10.1371/journal.pntd.0000902)
Supplement: Text S1 — Tekola Staging System (0.03 MB DOC) [file pntd.0000902.s002.doc]

**Appendix 1 – Tekola Clinical Staging System**

**Podoconiosis Staging Sheet (Podoconiosis-Endemic Areas)**

**Staging for Field Workers**

**Instructions**

The field worker is expected to look at and examine the right and left leg of each patient in turn and give a score to each leg separately.

• ‘Swelling’ here means a general increase in size of part of the foot or leg.

• ‘Reversible swelling’ here means a swelling that is not present when the patient first gets up in the morning and becomes more marked as the day advances.

• ‘Persistent swelling’ here means a swelling that is present all the time.

• ‘Knob or bump’ here means a discrete, hard lump that can be seen or felt to protrude from the rest of the foot or leg.

• ‘Ankle’ here means the level of the two ankle bones when the patient is standing.

• ‘Knee’ here means the level of the top of the knee cap when the patient is standing.

In addition to the numerical stage, the field worker should measure the greatest below-knee circumference and record the presence (M+) or absence (M)) of mossy changes. For example, if a patient’s right leg has irreversible below-knee swelling, nodules below the ankle, mossy changes around the heel and a circumference of 48 cm, the staging should be recorded as Stage 2, M+, 48.

**Stage 1. Swelling reversible overnight.** The swelling is not present when the patient first gets up in the morning.

**Stage 2. Below-knee swelling that is not completely reversible overnight; if present, knobs/bumps are below the ankle ONLY.** Persistent swelling that does not reach above the knee. If knobs or bumps are seen or felt, they are only present below the ankle, NOT above the ankle.

**Stage 3. Below-knee swelling that is not completely reversible overnight; knobs/bumps present above the ankle.** Persistent swelling that does not reach above the knee. Knobs or bumps can be seen or felt above the ankle as well as below.

**Stage 4. Above-knee swelling that is not completely reversible overnight; knobs/bumps present at any location.** Persistent swelling that is present above the knee. Knobs or bumps can be seen or felt at any place on the foot or leg.

**Stage 5. Joint fixation; swelling at any place in the foot or leg.** The ankle or toe joints become fixed and difficult to flex or dorsiflex. This may be accompanied by apparent shortening of the toes.

**Description for Health Professionals**

A more detailed and technical description of the changes that may be present at each stage is given, although the definitions for each stage remain the same. The stages represent severity of disease, and do not necessarily represent the disease process: it is possible, for example, for an individual to have Stage 5 disease but never to have had above-knee swelling. The following terms are used in the descriptions:

• Dermal nodules: elevated, non-translucent lesions >0.5 cm diameter, with width approximately equal to length.

• Dermal ridges: elevated lesions >0.5 cm width, with length greater than width.

• Dermal bands: palpable, but non-elevated ridges.

• Mossy changes: round or fusiform, either fluid-filled (and hence translucent) lesions, or papillomatous hyperkeratotic horny lesions giving the skin surface a rough velvet-like appearance.

**Stage 1. Swelling reversible overnight.** The swelling is not present when the patient first gets up in the morning. *Changes such as hyperpigmentation and nail dystrophy are unusual, but may be seen. The swelling is usually confined beneath the ankle.*

**Stage 2. Below-knee swelling that is not completely reversible overnight; if present, knobs/bumps are below the ankle ONLY.** *Persistent swelling that does not reach above the knee. If present, knobs or bumps do not extend beyond the ankle. The ‘knobs or bumps’ may take the form of dermal nodules, ridges or bands. Tourniquet-like effects may be observed at this stage or any subsequent stage, depending on the position of dermal ridges and nodules in relation to joints. Mossy changes may be apparent, but their presence depends on a range of factors including the use of plastic footwear. Interdigital maceration and hyperpigmentation are often present at this stage, and nail dystrophy almost always present.*

**Stage 3. Below-knee swelling that is not completely reversible overnight; knobs/bumps present above the ankle.** *Persistent swelling that does not reach above the knee. Dermal nodules, ridges or bands are seen or felt above the ankle. Tourniquet-like effects are frequently observed at this stage. Any of the other changes mentioned in Stage 2 may also be present.*

**Stage 4. Above-knee swelling that is not completely reversible overnight; knobs/bumps present at any location.** Persistent swelling that is present above the knee. *Any of the other changes mentioned in Stage 2 may also be present. In addition, signs of lymphectasia may be apparent, particularly on the thigh.*

**Stage 5. Joint fixation; swelling at any place in the foot or leg.** The ankle or interphalangeal joints becomes fixed and difficult to flex or dorsiflex. This may be accompanied by adhesion and fusion of the toe web spaces, making the toes appear short or indistinct. Sensation is preserved. *X-rays show tuft resorption and loss of bone density.*
